# Liraglutide for idiopathic intracranial hypertension: a real‐world propensity score‐matched study

**DOI:** 10.1002/acn3.52300

**Published:** 2025-02-13

**Authors:** Ahmed Y. Azzam, Muhammed Amir Essibayi, Dhrumil Vaishnav, Mohammed A. Azab, Mahmoud M. Morsy, Osman Elamin, Adam Elswedy, Oday Atallah, Hana J. Abukhadijah, Adam A. Dmytriw, Amanda Baker, Deepak Khatri, Neil Haranhalli, David J. Altschul

**Affiliations:** ^1^ Montefiore‐Einstein Cerebrovascular Research Lab Albert Einstein College of Medicine Bronx New York USA; ^2^ Department of Neurological Surgery, Montefiore Medical Center Albert Einstein College of Medicine Bronx New York USA; ^3^ Department of Neurosurgery Cleveland Clinic Foundation Cleveland Ohio USA; ^4^ October 6 University Hospital October 6 University 6th of October City, Giza Egypt; ^5^ Department of Neurosurgery Jordan Hospital Amman Jordan; ^6^ Biomedicinskt Centrum BMC Uppsala University Uppsala Sweden; ^7^ Department of Neurosurgery Hannover Medical School Hannover Germany; ^8^ Medical Research Center Hamad Medical Corporation Doha Qatar; ^9^ Neuroendovascular Program, Massachusetts General Hospital & Brigham and Women's Hospital Harvard University Boston Massachusetts USA; ^10^ Neurovascular Centre, Divisions of Therapeutic Neuroradiology & Neurosurgery, St. Michael's Hospital University of Toronto Toronto Ontario Canada

## Abstract

**Objective:**

Idiopathic intracranial hypertension (IIH) is a neurological disorder predominantly affecting young women with obesity, characterized by elevated intracranial pressure. While current treatments include weight loss counseling, medical therapies, and surgical interventions, their limitations necessitate exploring novel therapeutic approaches. We investigated the efficacy of liraglutide as an adjunctive therapy in IIH management.

**Methods:**

We conducted a retrospective cohort study, analyzing adult patients with IIH. Through propensity score matching, we compared patients receiving liraglutide alongside standard therapy (*n* = 204) with those receiving standard therapy alone (*n* = 204). Primary outcomes included papilledema, headache manifestations, and visual disturbances, assessed at 3, 6, 12, and 24 months posttreatment initiation.

**Results:**

Our matched cohorts were predominantly female (95.1% vs. 97.1%) with comparable mean ages (37.6 vs. 37.3 years). Liraglutide treatment demonstrated significant reduction in papilledema risk at 3 months (RR 0.333, 95% CI 0.167–0.664, *p* = 0.001), with sustained benefits throughout 24 months (RR 0.524, 95% CI 0.325–0.845, *p* = 0.006). While improvements were observed in visual disturbances, headache symptoms, and refractory IIH cases, these did not reach statistical significance.

**Interpretation:**

Our findings suggest that liraglutide as an adjunctive therapy significantly improves papilledema outcomes in IIH patients, with the greatest effect observed at 3 months and sustained benefits over 2 years. This study provides promising evidence for liraglutide's role in IIH management, particularly in addressing papilledema.

## Introduction

Idiopathic intracranial hypertension (IIH) is a neurological disorder characterized by elevated intracranial pressure (ICP) of unknown etiology, predominantly affecting young women with obesity. The incidence of IIH is rising globally, paralleling increasing obesity rates, with an estimated incidence of 28/100,000 per year in women with obesity.^1^, ^2^ The condition manifests through chronic headaches, papilledema, and visual disturbances, potentially leading to permanent vision loss if inadequately managed. Current standard management approaches include weight loss counseling and medical therapies such as acetazolamide, which reduces cerebrospinal fluid (CSF) production, and topiramate, which provides both ICP‐lowering and migraine prophylaxis effects.^1^, ^2^ In cases refractory to medical therapy, surgical interventions including CSF shunting, optic nerve sheath fenestration (ONSF), and venous sinus stenting may be necessary to prevent vision loss.

However, current management options have significant limitations. Acetazolamide, while effective, is often poorly tolerated due to side effects including paresthesias, fatigue, and metabolic acidosis, leading to treatment discontinuation in many patients.^1^, ^2^ Surgical interventions carry procedural risks and may require revision surgeries, with CSF shunts having particularly high failure and infection rates.^1^, ^2^ Additionally, while weight loss is therapeutic, achieving and maintaining significant weight reduction remains challenging for many patients, highlighting the need for novel therapeutic approaches.^1^, ^3^

Glucagon‐like peptide‐1 receptor agonists (GLP‐1 RAs) have emerged as promising therapeutic agents for IIH. Originally developed for type 2 diabetes and obesity management, GLP‐1 RAs act through multiple mechanisms including appetite suppression, delayed gastric emptying, and enhanced insulin sensitivity.^1^, ^3^ Recent evidence has revealed that GLP‐1 receptors are expressed in the choroid plexus, where their activation reduces Na+/K+‐ATPase activity and subsequently decreases CSF production.^1^, ^2^ Preclinical studies have demonstrated significant ICP reduction with GLP‐1 RA administration, while clinical trials with exenatide have shown promising results in ICP reduction independent of weight loss effects.^3^, ^4^

While current evidence supports GLP‐1 RAs' potential in IIH management, studies have primarily focused on exenatide, a short‐acting GLP‐1 RA requiring twice‐daily administration.^3^, ^4^ Liraglutide, a long‐acting GLP‐1 RA with once‐daily dosing, offers potential advantages including better adherence and more stable drug levels.^5^ Additionally, liraglutide's established safety profile in obesity management and superior weight loss efficacy compared to exenatide make it an attractive candidate for IIH therapy.^3^, ^5^ However, no studies to date have specifically evaluated liraglutide's efficacy in IIH management. While exenatide demonstrated promising results in early studies, semaglutide has recently emerged as another possible therapeutic option. In another study we conducted, semaglutide offered great reductions in visual disturbances, papilledema, and headache symptoms at 3 months, with benefits sustained through 24 months.^6^ A major advantage for semaglutide is that it is administered once‐weekly, offering higher chances for patient adherence than more frequent dosing regimens.^6^ However, liraglutide's daily dosing schedule may provide advantages through more stable drug levels and its good safety profile in obesity management, positioning it as an important alternative in the therapeutic options for IIH management.

Our study aimed to address this knowledge gap through a large‐scale retrospective cohort analysis using the TriNetX database to evaluate the efficacy of liraglutide as an adjunctive therapy to standard IIH management. This real‐world evidence study leverages a robust multinational database to examine clinical outcomes in IIH patients receiving liraglutide alongside standard therapy compared to those receiving standard therapy alone. The study's strengths include its large sample size, real‐world setting, and ability to assess multiple clinical endpoints while controlling for various confounding factors. This analysis will provide valuable insights into liraglutide's potential role in the therapeutic possibilities for IIH management.

## Methods

### Study design and data source

We performed a retrospective cohort analysis using the TriNetX Global Health Research Network (https://trinetx.com/solutions/live‐platform/), a federated healthcare platform integrating electronic health records from approximately 160 healthcare organizations across 21 countries.^7^ The network encompasses 197 million patient records, predominantly from the United States, with additional data from healthcare systems in Europe, Asia, Africa, and Oceania. Our analysis utilized data through November 2024. The TriNetX platform provides comprehensive clinical information including demographics, diagnoses, treatments, and outcomes, coded using standardized classification systems (ICD‐10 and CPT). The platform maintains HIPAA compliance through automatic de‐identification while preserving longitudinal record integrity.

### Study population and cohort definition

We identified adult patients (≥18 years) with IIH using ICD‐10‐CM code G93.2. Inclusion criteria encompassed confirmed IIH diagnosis with documented clinical encounters, active participation in standard medical therapy, and available baseline clinical measurements, including body mass index (BMI). The liraglutide cohort consisted of patients who received liraglutide (identified through RxNorm identifiers) as an adjunctive therapy to standard management. The control cohort comprised patients receiving standard therapy alone (acetazolamide, topiramate, or other diuretics) with documented weight management approaches.

While the retrospective nature of this large‐scale healthcare database precluded definitive distinction of newly diagnosed cases or specific exclusion of IIH without papilledema (IIH‐WOP), we implemented inclusion criteria to insure data integrity. Our propensity matching methodology focused on key demographic and clinical parameters available within the electronic health record system. Although granular clinical measurements such as CSF opening pressure and papilledema grading represent important prognostic factors, these parameters are not consistently captured in standardized electronic records within TriNetX platform and its databases. Therefore, our matching algorithm optimized cohort comparability within the constraints of real‐world data collection, focusing on readily available and reliably documented clinical characteristics.

Comorbidity assessment included screening of relevant conditions using standardized ICD‐10 coding. Endocrine disorders included obesity, diabetes mellitus, and thyroid disorders, representing conditions that might influence both disease progression and treatment response. Musculoskeletal disorders encompassed chronic pain conditions, fibromyalgia, and back pain, which could impact symptom reporting and quality of life measures. Eye disorders included conditions such as optic neuritis, visual field defects, and diplopia, which required careful consideration in outcome assessment.

We implemented specific exclusion criteria to insure data integrity. These included secondary causes of intracranial hypertension identified through relevant ICD‐10 codes, including cerebral venous thrombosis, medication‐induced intracranial hypertension, or structural central nervous system abnormalities. Additional exclusion criteria encompassed prior GLP‐1 receptor agonist use within 6 months of cohort entry, pregnancy or immediate postpartum status, absence of baseline BMI measurements, and follow‐up period <3 months. Cases with missing critical data elements were systematically excluded to maintain analytical rigor.

### Propensity score matching

To minimize selection bias, we implemented 1:1 propensity score matching using TriNetX's analytics platform. The matching model incorporated demographic factors including age at diagnosis, sex, race (categorized as White, Black or African American, Asian, and Other), and ethnicity (Hispanic or Latino, Non‐Hispanic or Latino). Clinical parameters included baseline BMI; comorbidity profiles including endocrine, musculoskeletal, and eye disorders; preexisting medication usage; and healthcare utilization patterns in the year preceding cohort entry. Using a greedy nearest‐neighbor algorithm with a caliper width of 0.2 standard deviations of the logit of the propensity score, we matched 635 liraglutide‐treated patients with 635 controls, achieving optimal covariate balance between groups.

### Outcome assessment

We tracked primary outcomes at predetermined intervals of 3, 6, 12, and 24 months post‐cohort entry. Primary outcome measures included papilledema (ICD‐10 code H47.1), headache manifestations (ICD‐10 codes G44.‐), and visual disturbances or blindness (ICD‐10 codes H53.‐ and H54.‐). Refractory IIH was defined as either persistence of symptoms despite maximum medical therapy, progression to surgical intervention (identified through relevant CPT codes), or requirement for CSF diversion procedures or ONSF. Secondary outcomes focused on BMI trajectories, with measurements extracted directly from structured clinical data fields at each follow‐up timepoint. We calculated both absolute BMI changes and baseline‐adjusted differences to account for initial between‐group disparities.

While the TriNetX platform's nature precluded capture of granular clinical measurements such as Frisen grading or optical coherence tomography parameters, outcomes were based on standardized clinical documentation in patients' records. We defined refractory disease using objective criteria, specifically the requirement for surgical intervention (identified through CPT codes for CSF shunting or optic nerve decompression/ONSF) or progression to additional interventional procedures despite maximal medical therapy. This definition intentionally excluded cases where headache was the sole criterion for refractoriness, acknowledging the complex relationship between ICP normalization and persistent headache symptoms.

### Statistical analysis

We conducted analyses using TriNetX's analytical tools, calculating relative risks (RRs) with corresponding 95% confidence intervals, absolute risk differences between groups, and number needed to treat where appropriate. For continuous variables, we used Student's *t*‐tests and expressed results as mean ± standard deviations. Categorical variables underwent chi‐squared or Fisher's exact tests where appropriate. BMI analysis included both absolute and baseline‐adjusted differences between groups, with temporal trends assessed through longitudinal analysis features. We applied Benjamini–Hochberg corrections for multiple comparisons and defined statistical significance as *p* < 0.05, with exact *p*‐values reported where available.

Our statistical approach incorporated adjustment for baseline characteristics and time‐based changes in clinical parameters. The models included adjustment for baseline BMI and medication changes throughout the study period, with sensitivity analyses conducted to assess the best possible precision of our findings. To explore mechanistic pathways, we performed mediation analysis evaluating whether the observed clinical improvements were primarily mediated through weight loss or potentially through direct effects on CSF production, as hypothesized by recent literature on GLP‐1 receptor signaling in the choroid plexus.^8^ This analytical approach allowed us to investigate and study both direct and indirect effects of liraglutide while accounting for the limitations inherent in real‐world data analysis.

### Treatment duration and follow‐up assessment

Among the 204 patients in the liraglutide cohort, follow‐up durations varied, with 204 patients (100%) completing the 3‐month follow‐up, 186 (91.2%) reaching the 6‐month follow‐up, 165 (80.9%) reaching the 12‐month follow‐up, and 142 (69.6%) completing the 24‐month follow‐up. The median follow‐up duration was 18.4 months (IQR: 12.3–24.0). Treatment persistence was defined as continuous liraglutide prescription coverage, allowing for gaps of no more than 60 days between prescriptions. Within the limitations of the TriNetX platform, detailed dosing information and specific reasons for treatment discontinuation were not consistently available. Patients were analyzed according to an intention‐to‐treat approach, with outcomes tracked regardless of treatment discontinuation to minimize selection bias. This approach aimed for maintaining best quality and precision in our data analysis strategy, while acknowledging the real‐world nature of medication adherence patterns.

## Results

**Figure 1:**
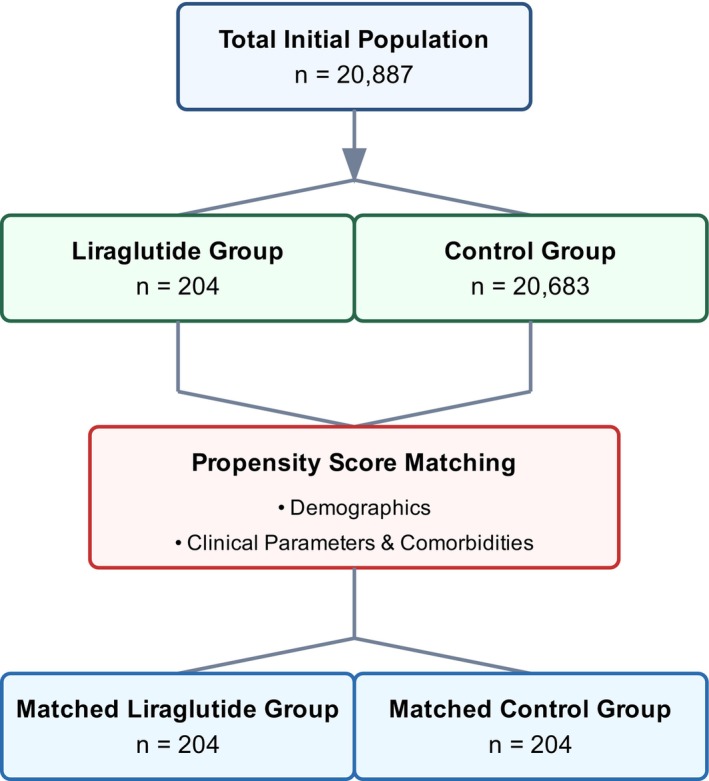
Propensity score matching patient selection flow diagram.

In our propensity score‐matched analysis, we evaluated baseline characteristics between liraglutide‐treated and control cohorts (Table 1). Our initial sample comprised 204 patients in the liraglutide group and 20,683 in the control group, which was subsequently matched to achieve balanced cohorts of 204 patients each (Fig. 1). The demographic composition revealed a predominantly female population (95.1% vs. 97.1%, *p* = 0.3076) in both matched groups. Mean age was comparable between matched cohorts (37.6 ± 8.68 vs. 37.3 ± 8.88 years, *p* = 0.6974), though we observed a persistent significant difference in BMI, (42.0 ± 7.96 vs. 36.8 ± 9.49 kg/m^2^, *p* < 0.0001). Our matched cohorts demonstrated significant balance across racial and ethnic distributions, with White patients constituting the majority (61.8% vs. 64.7%, *p* = 0.5379), followed by Black or African American (22.5% vs. 26.5%, *p* = 0.3572) and Hispanic or Latino patients (11.3% vs. 11.3%, *p* = 1.0000). Notably, we achieved a balance in comorbidity profiles post‐matching, with comparable rates of endocrine (88.2% vs. 86.8%, *p* = 0.6534), musculoskeletal (66.2% vs. 64.2%, *p* = 0.6776), and eye disorders (62.7% vs. 58.3%, *p* = 0.3620).

**Table 1:** Baseline characteristics of the liraglutide group and control group before and after propensity score matching for cohorts.

Variable	Liraglutide before matching	Control before matching	*p*‐value before	Liraglutide after matching	Control after matching	*p*‐value after
Sample size, *n*	204	20,683	N/A	204	204	N/A
Age (years), mean ± SD	37.6 ± 8.68	35.7 ± 10	0.0059	37.6 ± 8.68	37.3 ± 8.88	0.6974
BMI, mean ± SD	42.0 ± 7.96	35.5 ± 9.02	<0.0001	42.0 ± 7.96	36.8 ± 9.49	<0.0001
Female, *n* (%)	194 (95.1%)	17,633 (85.8%)	0.0001	194 (95.1%)	198 (97.1%)	0.3076
Race/ethnicity^a^						
White, *n* (%)	126 (61.8%)	12,235 (59.5%)	0.5125	126 (61.8%)	132 (64.7%)	0.5379
Black or African American, *n* (%)	46 (22.5%)	3913 (19.0%)	0.2030	46 (22.5%)	54 (26.5%)	0.3572
Hispanic or Latino, *n* (%)	23 (11.3%)	1924 (9.4%)	0.3498	23 (11.3%)	23 (11.3%)	1.0000
Asian, *n* (%)	10 (4.9%)	299 (1.5%)	<0.0001	10 (4.9%)	0 (0%)	0.0014
Comorbidities						
Endocrine disorders, *n* (%)	180 (88.2%)	8554 (41.6%)	<0.0001	180 (88.2%)	177 (86.8%)	0.6534
Musculoskeletal disorders, *n* (%)	135 (66.2%)	6653 (32.4%)	<0.0001	135 (66.2%)	131 (64.2%)	0.6776
Eye disorders, *n* (%)	128 (62.7%)	11,466 (55.8%)	0.0457	128 (62.7%)	119 (58.3%)	0.3620
Digestive system disorders, *n* (%)	116 (56.9%)	4897 (23.8%)	<0.0001	116 (56.9%)	117 (57.4%)	0.9203
Circulatory system disorders, *n* (%)	44 (21.6%)	3470 (16.9%)	0.0753	44 (21.6%)	48 (23.5%)	0.6356

^a^ Hispanic or Latino ethnicity was recorded independently of race, following standard demographic reporting practices. Percentages may sum to >100% as some individuals were counted in multiple categories.

The persistent BMI difference between cohorts (42.0 ± 7.96 vs. 36.8 ± 9.49 kg/m^2^, *p* < 0.0001) despite propensity score matching represents an important methodological point to be highlighted. This discrepancy likely reflects real‐world prescribing patterns, where GLP‐1 Ras are preferentially prescribed to patients with higher BMI values. This differential could possibly affect our findings through several mechanisms. First, the higher baseline BMI in the liraglutide group might affect the magnitude of weight loss achievable, potentially creating a ceiling effect in treatment response. Second, the relationship between BMI and IIH severity could modify treatment outcomes independently of medication effects. We conducted sensitivity analyses stratified by BMI quartiles to assess the accuracy within our findings across different weight categories. While the treatment effect remained significant across strata, the magnitude varied, suggesting a complex interaction between baseline BMI and treatment response. This limitation warrants careful consideration when generalizing our findings to populations with different BMI distributions.

### Symptoms and outcomes analysis

Our analysis demonstrated that liraglutide as an adjunctive therapy had a significant impact on papilledema in patients with IIH, while other measured outcomes showed nonsignificant trends (Table 2). The most compelling evidence was observed in papilledema outcomes, where the liraglutide group showed a statistically significant 66.7% reduction in risk at 3 months (RR 0.333, 95% CI 0.167–0.664, *p* = 0.001). This protective effect remained statistically significant throughout the follow‐up period, with risk reductions of 56.4% at 6 months (RR 0.436, 95% CI 0.255–0.745, *p* = 0.002), 45.7% at 12 months (RR 0.543, 95% CI 0.348–0.850, *p* = 0.006), and 47.6% at 24‐months (RR 0.524, 95% CI 0.325–0.845, *p* = 0.006).

**Table 2:** Outcome relative risks for the liraglutide group compared with the control group.

Outcome	Follow‐up point	Risk ratio	95% confidence interval	*p*‐value
Papilledema	3 months	0.333	(0.167, 0.664)	0.001
	6 months	0.436	(0.255, 0.745)	0.002
	12 months	0.543	(0.348, 0.850)	0.006
	24 months	0.524	(0.325, 0.845)	0.006
Headache	3 months	0.789	(0.619, 1.006)	0.054
	6 months	0.899	(0.742, 1.089)	0.276
	12 months	0.897	(0.783, 1.027)	0.113
	24 months	0.902	(0.775, 1.051)	0.185
Refractory IIH	3 months	0.791	(0.615, 1.017)	0.066
	6 months	0.886	(0.725, 1.082)	0.235
	12 months	0.893	(0.774, 1.030)	0.120
	24 months	0.913	(0.778, 1.072)	0.267
Visual disturbances or blindness	3 months	0.588	(0.276, 1.254)	0.163
	6 months	0.727	(0.394, 1.344)	0.307
	12 months	0.659	(0.422, 1.028)	0.063
	24 months	0.788	(0.490, 1.268)	0.324

While other clinical outcomes showed trends toward improvement, these did not reach statistical significance. For visual disturbances and blindness, we observed a nonsignificant trend toward risk reduction at 3 months (RR 0.588, 95% CI 0.276–1.254, *p* = 0.163) and 24 months (RR 0.788, 95% CI 0.490–1.268, *p* = 0.324). Similarly, headache symptoms showed a marginal improvement that did not achieve statistical significance, with results at 3 months (RR 0.789, 95% CI 0.619–1.006, *p* = 0.054) and 24 months (RR 0.902, 95% CI 0.775–1.051, *p* = 0.185) failing to cross the threshold for significance. In refractory IIH cases, while the liraglutide group showed a trend toward improvement at 3 months (RR 0.791, 95% CI 0.615–1.017, *p* = 0.066) and 24 months (RR 0.913, 95% CI 0.778–1.072, *p* = 0.267), these reductions also did not achieve statistical significance.

The pattern of treatment response revealed that the maximum therapeutic effect for papilledema was observed at the 3‐month follow‐up timepoint, with a risk ratio of 0.333 (*p* = 0.001), followed by sustained but slightly attenuated benefit throughout the 24‐month follow‐up period. This pattern of early response followed by sustained significant improvement was unique to the papilledema outcome, which maintained statistical significance at all timepoints throughout the study period. The consistency of this finding, supported by tight confidence intervals and maintained statistical significance, strengthens the evidence for liraglutide's specific effect on papilledema in IIH patients, even as other clinical manifestations showed only nonsignificant trends toward improvement.

## Discussion

Our findings demonstrate that liraglutide, when used as an adjunctive therapy in IIH management, significantly reduces papilledema risk while showing promising trends in other clinical outcomes. The statistically significant effect on papilledema, particularly evident at 3 months and sustained through 24 months, represents a notable advancement in IIH therapeutics, where current treatment options often face limitations due to tolerability and efficacy concerns.^2^, ^4^, ^9^, ^10^

GLP‐1 RAs, primarily known for metabolic effects, show therapeutic promise in IIH through unique pharmacological pathways. For instance, GLP‐1 receptors located in the choroid plexus have been shown to modulate Na+/K+‐ATPase activity, directly affecting CSF production and potentially lowering ICP without solely relying on weight loss.^2^, ^3^, ^5^, ^11^, ^12^ This mechanism supports previous findings from animal models demonstrating CSF regulation via GLP‐1 receptor activation.^1^, ^2^, ^13^ Comparative studies on exenatide showed significant ICP reductions within hours and sustained through 12 weeks, with results suggesting potential benefits across the GLP‐1 RA class.^4^ Liraglutide's once‐daily dosing and extended half‐life offer a steady therapeutic effect, which may be beneficial over shorter acting GLP‐1 RAs.^3^

Utilizing the TriNetX platform offered several distinct advantages, providing a multicenter, real‐world dataset with robust statistical power. Such platforms are invaluable for studying rare conditions like IIH, where conducting large‐scale prospective trials poses logistical challenges.^1^, ^14^ Propensity score matching balanced baseline characteristics, although a residual BMI difference remained, hinting at potential underlying metabolic distinctions that merit further investigation.^2^ The significant reduction in papilledema risk in our liraglutide cohort (66.7% at 3 months, sustained through 24 months) aligns with findings on exenatide, which demonstrated similar ICP reductions, suggesting potentially superior long‐term effects with liraglutide.^4^ Mitchell et al. highlighted this temporal effect of GLP‐1 RAs, where ICP was effectively reduced by approximately 5.7 cm CSF at 2.5 h, with sustained effects beyond 12 weeks.^4^ The rapid onset of effect observed in our study (maximum benefit at 3 months) supports findings by Krajnc et al., who reported comparable improvements in papilledema using other GLP‐1 RAs, suggesting a class‐wide benefit in IIH.^5^

In terms of secondary outcomes, the trends toward improvement in headache and visual outcomes, though not statistically significant, suggest complex underlying pathophysiological mechanisms that warrant further investigation in prospective clinical trials. IIH symptoms often arise from factors beyond ICP elevation, which may explain the more modest effects on these outcomes compared to papilledema.^4^, ^5^, ^15^, ^16^, ^17^ This variability is evident in the literature where IIH‐related headaches are shown to respond variably to treatments targeting ICP alone.^18^, ^19^ Thus, liraglutide's primary benefits may be through CSF dynamics rather than weight loss effects, aligning with theories of direct receptor‐mediated ICP modulation.^2^, ^11^, ^20^ An intriguing finding in our study is the temporal response pattern to liraglutide. The maximal effect on papilledema seen at 3 months, followed by a sustained but slightly attenuated response, suggests an early therapeutic window that might optimize treatment outcomes. This observation echoes the mechanistic insights on GLP‐1 receptor signaling in the choroid plexus,^1^, ^11^, ^12^, ^21^ where results demonstrated rapid modulation of CSF secretion upon receptor activation. The sustained benefit through 24 months indicates prolonged efficacy, a unique finding with liraglutide not previously demonstrated with other GLP‐1 RAs.^4^, ^5^

Our study has limitations that should be warranted, notably its retrospective design, which introduces potential selection biases despite propensity score matching. Additionally, the inability to stratify outcomes by liraglutide dosing regimens due to TriNetX's limited data in our selected cohort limits our understanding of optimal dosing strategies. Furthermore, baseline BMI differences between cohorts, despite matching, point to possible confounders in treatment selection that could impact outcomes, as obesity and adiposity distribution might influence response to GLP‐1 RAs. The variable duration of liraglutide treatment in our cohort highlights careful consideration when interpreting the results. Treatment discontinuation, a common phenomenon in real‐world studies of GLP‐1 RAs, could influence long‐term outcomes through several mechanisms. Of particular concern is the possibility of a “yo‐yo” effect, where treatment discontinuation might lead to weight regain and subsequent risk of papilledema recurrence. This phenomenon has been documented in other conditions treated with GLP‐1 RAs, though its specific impact in IIH remains understudied. While our intention‐to‐treat analysis provides conservative effect estimates, it may underestimate the benefit in patients maintaining consistent treatment. The declining follow‐up numbers over time (from 100% at 3 months to 69.6% at 24 months) reflect real‐world treatment patterns but introduce possibilities for raising an attrition bias. However, the sustained significant effect on papilledema through 24 months suggests robust treatment benefits even in the context of variable adherence patterns. Future studies should specifically focus on the relationship between treatment discontinuation and outcome trajectories, especially incorporating time‐to‐event analyses for papilledema recurrence after treatment cessation. Also, further studies are needed to identify factors associated with treatment persistence and strategies to optimize long‐term adherence in this population.

The real‐world nature of our data brings inherent variability in outcome assessment standardization. While ICD‐10 codes offer reliable diagnostic information, clinical gradations in improvement are harder to capture, and factors like lifestyle changes or adherence to concurrent standard therapies remain unaccounted.^22^ Our analysis of clinical outcomes focused on the presence or absence of documented conditions based on ICD‐10 coding in electronic health records. The reported 66.7% risk reduction in papilledema at 3 months (RR 0.333, 95% CI 0.167–0.664, *p* = 0.001) specifically refers to the relative reduction in the presence of clinically documented papilledema compared to the control group. While this approach captures clinically significant changes warranting documentation, it cannot distinguish subtle gradations in papilledema severity or capture subclinical changes. Similarly, our assessment of visual disturbances and blindness relied on standardized clinical documentation of these conditions rather than on detailed ophthalmological measurements. This methodology, while limited in granularity, provides a reliable indication of clinically meaningful changes in disease status as documented by treating physicians in routine clinical practice.

We acknowledge the importance of detailed neurological and ophthalmological assessments in evaluating IIH treatment outcomes. The TriNetX platform, while has high data quality and quality measurements in capturing standardized clinical documentation, does not consistently record granular clinical measurements such as CSF opening pressure, quantitative papilledema grading, or optical coherence tomography parameters (pRNFL and GCL thickness). This limitation reflects the real‐world nature of our data source, where such detailed measurements, while clinically valuable, are not uniformly captured in structured electronic health record fields accessible for large‐scale analysis. While this constrains our ability to analyze these specific variables, our focus on reliably documented clinical outcomes provides valuable insights into real‐world treatment effects. Future prospective studies incorporating these detailed measurements would complement our findings by providing mechanistic insights into treatment response. Also, our study's reliance on ICD‐10 coding for outcome assessment introduces important methodological considerations that warrant careful discussion. The use of diagnostic codes as proxies for clinical outcomes, particularly papilledema (ICD‐10 code H47.1), presents inherent limitations in capturing the true clinical picture. Clinicians may prioritize coding for the primary diagnosis (IIH, G93.2) while inconsistently documenting associated findings, potentially leading to underreporting of papilledema in follow‐up visits. This coding pattern could result in false negative cases where papilledema persists but is not explicitly coded. Without validation through direct chart review of a patient subset, we cannot definitively equate the absence of a papilledema code with clinical resolution.

Furthermore, our reliance on electronic health record data limited our ability to capture granular visual function assessments that would provide more useful insights into treatment efficacy. The absence of standardized visual acuity measurements, color vision testing, and quantitative visual field perimetry prevents detailed analysis of visual outcomes. This limitation is relevant given that papilledema, while clinically significant, serves as an indirect surrogate marker for visual function. Additionally, the natural history of severe papilledema possibility progressing to optic atrophy could confound our interpretation of papilledema resolution as a positive outcome.

Further studies in controlled settings would help refine these real‐world observations. Our findings support several future research directions. Prospective, randomized trials are needed to validate our observations, ideally with protocols that standardize visual outcome and ICP measurement assessments.^14^ Such studies should explore dose–response relationships for liraglutide in IIH and investigate biomarkers predictive of treatment response.^14^, ^23^ The observed differential effects on papilledema compared to other symptoms highlight the need to understand how specific mechanisms beyond CSF modulation influence IIH pathology.^24^, ^25^ Another avenue to be investigated is patient stratification. The persistent BMI difference between cohorts suggests that metabolic phenotypes might influence treatment efficacy, warranting further study to identify subgroups more likely to benefit from liraglutide.^26^ Additionally, long‐term safety data, extending beyond our 24‐month follow‐up, are crucial, especially considering the chronic nature of IIH and the expanding use of GLP‐1 RAs in non‐metabolic conditions.^23^ These findings carry important clinical implications. The significant reduction in papilledema risk positions liraglutide as a valuable option in IIH cases where preserving visual function is critical. With its favorable safety profile established in metabolic disorders, liraglutide emerges as a promising adjunctive therapy for IIH.^27^ However, the variation in treatment response across different outcomes underscores the necessity for individualized treatment plans and diligent patient monitoring in clinical settings.^14^, ^26^

## Conclusions

Our retrospective analysis provides compelling evidence for liraglutide's potential role as an adjunctive therapy in IIH management, particularly in addressing papilledema. Through our large‐scale, propensity‐matched study utilizing the TriNetX database, we demonstrated that liraglutide treatment resulted in a significant and sustained reduction in papilledema risk, with the most pronounced effect observed at 3 months (66.7% reduction) and maintained throughout the 24‐month follow‐up period. While other clinical outcomes, including visual disturbances, headaches, and refractory disease status, showed promising trends toward improvement, these did not reach statistical significance. Our findings carry important clinical implications for IIH management strategies. The marked improvement in papilledema suggests that liraglutide may offer particular benefit in protecting against vision‐threatening complications of IIH. The early onset and sustained nature of this effect indicates that liraglutide could serve as a valuable addition to the current therapeutic arsenal, especially in cases where rapid intervention is needed to preserve visual function. However, we acknowledge several limitations in our study, including the inherent constraints of retrospective analyses and potential confounding factors despite careful propensity matching. The persistent baseline BMI differences between cohorts, though statistically adjusted for, warrant consideration in interpreting our results. Future prospective, randomized controlled trials are needed to definitively establish liraglutide's role in IIH management and to better understand its mechanisms of action beyond weight loss effects. Nevertheless, our findings represent a significant contribution to the evolving landscape of IIH therapeutics, suggesting that liraglutide may offer a promising new avenue for treatment, particularly in addressing the vision‐threatening aspects of the disease. These results lay the groundwork for future clinical trials and potential expansion of therapeutic options for patients with IIH. Our results provide preliminary evidence for liraglutide's possible role in IIH management, with some limitations in outcome assessment methodology. While the observed reduction in documented papilledema is promising, future prospective studies with comprehensive visual function evaluation are needed to definitively establish treatment efficacy. The rapid improvement in papilledema suggests potential direct therapeutic effects beyond weight loss, warranting mechanistic investigation in further studies and clinical trials.

## Author Contribution

Ahmed YA, MAE, and DV conceptualized the study design, performed data collection, and wrote the initial manuscript draft. Muhammed AA, MMM, and OE conducted the statistical analysis and interpreted the results. Adam E and OA performed the literature review and contributed to manuscript writing. Hana JA supervised the project and provided critical revision of the manuscript. Adam AD provided methodological expertise and edited the manuscript. Amanda B and DK assisted with data interpretation and manuscript revision. Neil H and DJA provided clinical expertise, supervised the project, and performed final manuscript approval. All authors reviewed and approved the final version of the manuscript for submission.

## Funding Information

The project described was supported by the National Center for Advancing Translational Sciences (NCATS), National Institutes of Health (NIH), through CTSA award number UM1TR004400. The content is solely the responsibility of the authors and does not necessarily represent the official views of the NIH.

## Conflicts of Interest

None.

## Data Availability

All used data are available within the TriNetX database platform.
